# Upper limb progression in Duchenne muscular dystrophy: Insights from a 36-month longitudinal study using the PUL 20

**DOI:** 10.1177/22143602251355318

**Published:** 2025-09-18

**Authors:** Giorgia Coratti, Marika Pane, Sophia Paolucci, Luca Bello, Adele D’Amico, Angela Berardinelli, Michela Catteruccia, Giacomo De Luca, Riccardo Masson, Riccardo Zanin, Roberta Battini, Claudia Dosi, Silvia Frosini, Alice Gardani, Bianca Buchignani, Anna Capasso, Federica Ricci, Gianpaolo Cicala, Ilaria Cavallina, Enrica Rolle, Tiziana Enrica Mongini, Valeria Ada Sansone, Emilio Albamonte, Antonella Pini, Melania Giannotta, Chiara Panicucci, Riccardo Not, Claudio Bruno, Vincenzo Nigro, Esther Picillo, Elena Pegoraro, Eugenio Mercuri, On behalf of the Italian DMD group

**Affiliations:** 1 Pediatric Neurology, 60234 Università Cattolica del Sacro Cuore, Rome, Italy; 2 Centro Clinico Nemo, Neuropsichiatria Infantile, Fondazione Agostino Gemelli IRCCS, Rome, Italy; 3 Neurology Unit, 18624 Azienda Ospedale Padova Università Padova, Padua, Italy; 4 Department of Neurosciences, Unit of Neuromuscular and Neurodegenerative Disorders, 9342 Bambino Gesù Children's Hospital, IRCCS, Rome, Italy; 5 C. Mondino Foundation, Pavia, Italy; 6 Developmental Neurology Unit, 9328 Fondazione IRCCS Istituto Neurologico Carlo Besta, Milan, Italy; 7 Department Neuroscience, 9357 IRCCS Stella Maris Foundation, Pisa, Italy; 8 Department Clinical and Experimental Medicine, University of Pisa, Pisa, Italy; 9 Department of Translational Research and of New Surgical, Medical Technologies Pisa University, Pisa, Italy; 10 Child Neuropsychiatry Unit, Department of Public Health and Paediatric Sciences, 9314 University of Turin, Turin, Italy; 11 Neuromuscular Unit, Department of Neurosciences, 9314 University of Turin, Turin, Italy; 12 Neurorehabilitation Unit, The NEMO Center in Milan, University of Milan, 9338 ASST Niguarda Hospital, Milan, Italy; 13 Pediatric Neuromuscular Unit, 419170 IRCCS Institute of the Neurological Sciences of Bologna, Bologna, Italy; 14 Department of Neuroscience, Rehabilitation, Ophtalmology, Genetics, Maternal and Child Health, Center of Translational and Experimental Myology, University of Genova, 18572 IRCCS Istituto Giannina Gaslini, Genova, Italy; 15 Medical Genetics and Cardiomyology Unit, Department of Precision Medicine, 507855 Università degli Studi della Campania “Luigi Vanvitelli”, Napoli, Italy; 16 TIGEM, Pozzuoli, Italy

**Keywords:** upper limb function, Duchenne muscular dystrophy, performance of upper limb, longitudinal study

## Abstract

**Introduction:**

Duchenne muscular dystrophy (DMD) is a progressive disorder. This study evaluates upper limb function in DMD patients using the Performance of Upper Limb 2.0 (PUL 2.0) over 36-months.

**Methods:**

Data were collected between 2011 and 2024. Patients with at least 36 months of follow-up were included. Mixed-effects models accounting for repeated measures evaluated 36-month PUL 2.0 changes by entry item and ambulatory status. The entry item assesses the overall upper limb function of the patient. Ambulant patients were defined as those able to walk 10 meters independently, transitioning patients as those who lost ambulation during the duration of the study and non-ambulant as those who had already lost ambulation at baseline.

**Results:**

A total of 219 patients provided 684 paired 36-month assessments. Ambulatory status significantly affected total, shoulder, elbow, and distal scores at baseline. The largest 36-month decline in total scores was found in the 58 transitioning patients (11.62 points, 95%CI = −12.40, 10.84), followed by non-ambulant and ambulant subgroups (n = 116 and n = 86 respectively). The largest declines were seen in patients with baseline entry score of 4 (−11.97, 95% CI = −13.48, −10.46) and 5 (−11.55, 95% CI = −12.46, −10.63), with smaller declines for other entry scores.

**Conclusions:**

The 36-month analysis confirms a clear trend of functional decline across time points, with the transitioning group exhibiting the greatest changes in upper limb function. These findings provide valuable insights for designing trials and offer a reference for long-term comparison of treatment efficacy in both experimental and real-world setting.

## Introduction

Duchenne muscular dystrophy (DMD) is a rare genetic neuromuscular disorder characterized by progressive muscle degeneration and weakness, primarily affecting boys due to its X-linked recessive inheritance. With an estimated prevalence of 1 in 5000 to 1 in 6000 live male births, DMD results from mutations in the dystrophin gene (DMD), which encodes the dystrophin protein, essential for maintaining muscle fiber stability during contraction.^1^ The absence or deficiency of dystrophin leads to the disruption of muscle fibers, which are gradually replaced by fibrous adipose tissue, accelerating disease progression.^2,3^

There is a progressive involvement of skeletal muscles with a proximal to distal gradient of weakness initially affecting mainly lower limbs, with loss of ambulation around puberty, and subsequent loss of function in the upper limbs.^4–6^ The advent of clinical trials in non-ambulant boys and young adults has highlighted the need to identify reliable tools for assessing upper limb function and to collect natural history data.^7–13^

The Performance of Upper Limb (PUL 2.0) scale is a functional assessment tool specifically designed to evaluate upper limb performance in DMD patients, aiming to monitor disease progression and track the gradual loss of proximal to distal functionality. Applicable to both ambulant and non-ambulant patients, the PUL 2.0 is organized into three anatomical domains: proximal (shoulder function), middle (elbow functionality), and distal (wrist and finger function), therefore allowing to follow the typical proximal to distal progression of upper limb involvement.^14,15^

Several papers have reported how the PUL 2.0 scores can detect and quantify the functional decline in both ambulant and non-ambulant, suggesting that the decline is not uniform.^16–20^ The involvement of upper limbs is relatively small in young DMD boys soon after diagnosis, while between the age of 9 and 16 years there is a marked decline in PUL 2.0 scores reflecting the increasing weakness in shoulder and elbow domains and, at a later age, also of the distal one.^17^ The scale has been used in several natural history studies reporting the association between PUL 2.0 scores and different clinical variables, such as ambulant status, and with patient reported outcomes or imaging findings.^21–28^

These studies have provided evidence of the variability of progression of the PUL 2.0 scores over time and have highlighted the need for stratification by key clinical factors—such as ambulatory status and baseline function to identify more defined trajectories of progression in individual subgroups.This is of particular relevance at the time of designing clinical trials using the PUL as an outcome measure for stratification and powering of the study.

Furthermore, as recent long term follow up data from clinical trials in DMD have highlighted that better results are often observed with increasing duration of the study, there has been increasing attention to the need for long term natural history data. So far, the number of longitudinal studies published using the PUL 2.0 is limited to one or two years follow up data.^16,17,20^ Further expanding the availability of long-term longitudinal data will facilitate our understanding of the long term natural progression in untreated patients and provide a critical baseline for long-term comparisons with treated cohorts. This longitudinal study aims (i) to evaluate PUL 2.0 scores in a cohort of pediatric and adult DMD patients over a 36-month period, analyzing functional progression across the total score and specific domains (shoulder, elbow, and wrist); (ii) to stratify the cohort according to different variables including PUL 2.0 entry item and functional status.

## Methods

The cohort included data collected between September 2011 and March 2024, including all patients with a confirmed genetic diagnosis of DMD, with an age ≥7 years (±3 months) and naïve from experimental pharmacological treatments.

The study received approval from the ethics committees of 15 national tertiary centers: Catholic University (Rome), Centro Clinico Nemo (University of Milan, Milan), IRCCS Eugenio Medea Bosisio-Parini (Bosisio-Parini), IRCCS Istituto Giannina Gaslini (Genoa), University of Messina (Messina), IRCCS Ospedale San Raffaele (Milan), Fondazione IRCCS Istituto Neurologico Besta (Milan), Fondazione IRCCS Ca’ Granda – Ospedale Maggiore Policlinico (Milan), University of Naples (Naples), Ospedale Bambino Gesù (Rome), University of Padua (Padua), Istituto Mondino (Pavia), University of Turin (Turin), the Neuromuscular Pediatric Unit, IRCCS Istituto delle Scienze Neurologiche di Bologna (Bologna) and), IRCCS Stella Maris Foundation, Calambrone, Pisa. Informed consent was obtained from all participants or their legal guardians.

### Performance of upper limb

The Performance of Upper Limb (PUL 2.0) is a functional scale designed to assess upper limb performance in patients with Duchenne muscular dystrophy (DMD), applicable to both ambulant and non-ambulant patients.^15,29^ Initially, the scale was developed as PUL 1.2 and after a few years revised to PUL 2.0.^29^

PUL 2.0 is divided into three domains, each corresponding to an anatomical region: the proximal domain assesses shoulder function, the middle domain evaluates elbow function, and the distal domain assesses wrist and finger function. All items simulate movements used in daily activities, thereby also providing an assessment of the patient's autonomy.

PUL 2.0 consists of 22 items distributed as follows: 6 items for the proximal region, 9 for the middle region, and 7 for the distal region. The entry item (Item A) is based on the Brooke scale, with a score ranging from 0 (no hand function) to 6 (complete shoulder abduction without compensatory movements). However, this score is not added to the subsequent items, as the movement is assessed in greater detail in the following tests. If the patient scores below 3, only the items related to the middle and distal regions are administered, as proximal ones would be unfeasible. The maximum score in PUL 2.0 is 42, with 12 points for the proximal level, 17 for the middle level, and 13 for the distal level. A copy of the PUL 2.0 scoresheet and manual can be accessed at (https://www.pod-nmd.org/assessment/pul/).

Patients were included regardless of their ambulatory status and were classified as follows: Ambulant patients were those able to walk 10 meters independently; transitioning patients were those who lost the ability to walk during the study; and non-ambulant patients were those who had already lost ambulation at baseline.

The following functional tools were also used to further characterize ambulant patients.

### North star ambulatory assessment

The North Star Ambulatory Assessment (NSAA) is a clinician-administered, functional scale designed to evaluate motor abilities in ambulant boys with Duchenne muscular dystrophy.^30^ It includes 17 items that assess activities such as standing, walking, climbing, and rising from the floor, with each item scored on a 3-point scale based on performance. The NSAA provides a standardized way to monitor disease progression and response to therapy in both clinical and research settings (https://www.pod-nmd.org/assessment/nsaa/).

### 6-min walk test

The 6-Minute Walk Test (6MWT) is a widely used functional assessment that measures the distance an individual can walk in six minutes on a flat, hard surface, along a 25-meter straight path, walking as fast as possible without running.^31^ In Duchenne muscular dystrophy (DMD), it serves as a key outcome measure to evaluate endurance, ambulatory function, and disease progression in ambulant patients (https://www.pod-nmd.org/assessment/6-minute-walk-test/).^32–34^

### Statistical analysis

A longitudinal dataset with paired visits over a 36-month period was analyzed to assess differences in PUL 2.0 changes across different variables.

Data included PUL 2.0 total score and subdomains scores, age at evaluation, entry item, ambulatory status (ambulant, transitioning, non-ambulant), steroids frequency (daily, intermittent, naïve) and type (naïve, prednisolone/prednisone, corticosteroids).

When a patient had a longer follow-up period, all eligible 36-month follow-up segments were considered for the analysis. However, for each specific starting age, only one 36-months interval per patient was included to prevent duplication due to overlapping intervals beginning at the same age. This approach was adopted to ensure the independence of data points and avoid bias in the analysis.

This procedure was implemented to ensure that each observation contributed independently to the analysis and to prevent bias due to repeated measurements from overlapping timeframes.

Only patients with at least 36 months of follow-up and complete data at baseline, 12- and 24-months were analyzed.

Continuous variables were reported as either mean and standard deviation or 95% confidence intervals (SD/95% CI), depending on their distribution, while categorical variables were reported as median and interquartile range (IQR) and/or frequencies and percentages. Continuous variables include those that can take any value within a range (e.g., age, total score) and are treated as such for analysis. Categorical variables, on the other hand, include those that represent distinct groups or categories (e.g., ambulatory status groups, PUL 2.0 entry item).

To accommodate the multiple assessments per participant, means and 95% confidence intervals for baseline values estimated using a linear mixed-effects model with a random intercept for each patient. This approach adjusts for the correlation of measurements within individuals. Pairwise comparisons were adjusted using the Tukey-Kramer method.

In order to better delineate the functionality of ambulant and transitioning groups, a linear mixed-effects model was employed to compare 6MWT and NSAA scores between ambulant and transitioning groups at the first assessment of each 36-month follow-up segment. To account for repeated measures within individuals, patient ID was included as a random effect, while group (ambulant vs. transitioning) was included as a fixed effect. Model estimates are presented with 95% confidence intervals.

Additionally, a linear mixed-effects model was also used to evaluate changes over time in PUL 2.0 total scores. Random effects included a random intercept for each participant. A significance threshold of 0.05 was applied to all statistical tests. Data processing and analyses were conducted using RStudio.

## Results

Two hundred nineteen patients had at least one pair of assessments at 36 months, for a total of 684 paired assessments. Baseline characteristics can be found in Table 1.

**Table 1.** Baseline characteristics of the cohort subdivided in ambulatory status. Key to table: (n = number of patients; number of assessments).

		Whole cohort (n = 219; 684)	Non ambulant (n = 75; 333)	Transitioning (n = 58; 151)	Ambulant (n = 86; 200)
Age	Mean (SD)	12.9 (4.78)	15.8 (4.80)	10.5 (2.40)	9.74 (2.65)
	Median [Min, Max, IQR]	11.8 [6.76, 30.2, 6.00]	14.8 [6.83, 30.2, 6.70]	10.3 [6.80, 21.7, 3.16]	9.10 [6.76, 19.9, 2.62]
Mutations	Deletions (N, %)	459 (67.1%)	245 (73.6%)	96 (63.6%)	118 (59.0%)
	Duplications (N, %)	51 (7.5%)	17 (5.1%)	21 (13.9%)	13 (6.5%)
	Point mutations (N, %)	174 (25.4%)	78 (21.3%)	34 (49.7%)	69 (47.5%)
Steroids type and frequency	Corticosteroids – Daily	429 (62.7%)	181 (54.4%)	107 (70.9%)	141 (70.5%)
	Corticosteroids – Intermittent	169 (24.7%)	83 (24.9%)	34 (22.5%)	52 (26.0%)
	Naïve	51 (7.5%)	44 (13.2%)	3 (2.0%)	4 (2.0%)
	Prednisone – Daily	29 (4.2%)	19 (5.7%)	7 (4.6%)	3 (1.5%)
	Prednisone – Intermittent	6 (0.9%)	6 (1.8%)	0 (0%)	0 (0%)
PUL 2.0 score	Total – Mean (95% CI)	32.71 (31.70–33.72)	24.35 (23.11–25.58)	35.58 (34.27–36.89)	38.19 (36.75–39.63)
	Shoulder – Mean (95% CI)	7.66 (7.29–8.03)	3.47 (2.89–4.05)	9.02 (8.36–9.67)	10.49 (9.79–11.18)
	Elbow – Mean (95% CI)	13.50 (12.98–14.02)	9.90 (9.11–10.68)	14.82 (13.98–15.67)	15.78 (14.86–16.70)
	Distal – Mean (95% CI)	11.58 (11.35–11.81)	10.74 (10.41–11.07)	11.80 (11.46–12.14)	12.19 (11.82–12.57)
Number of 36-month assessments included per patient	Mean (SD)	3.12 (1.65)	2.87 (1.52)	1.74 (0.84)	2.33 (1.44)
	Median [Min, Max, IQR]	3 [1, 8, 2]	3 [1, 8, 2]	1 [1, 4, 1]	2 [1, 6, 2]

To better delineate the profile of transitioning assessments, statistical analysis was conducted on the concomitant baseline data for the PUL 2.0 and 6MWT. Data were available for 175 ambulant assessments and 141 transitioning assessments (88% and 93%, respectively). The analysis showed a statistically significant difference between the two groups. Specifically, individuals in the transitioning group walked 96.92 meters less on the 6MWT (Mean = 314.06) compared to those in the ambulant group (Mean = 432.12), t(303.45) = −9.45, p < .001.

The same analysis was applied to NSAA. Concomitant PUL 2.0 and NSAA baseline data were available for 152 ambulant and 130 transitioning assessments (76% and 86% respectively). The analysis revealed a statistically significant difference between the two groups. Specifically, transitioning individuals scored 7.40 points lower on the NSAA (M = 16.57) compared to ambulant individuals (Mean = 25.83), t(276.89) = −9.14, p < .001.

### Baseline PUL 2.0 scores

A linear mixed-effects model, accounting for repeated measures (n = 684; 219 individuals), showed a significant effect of ambulatory status on baseline total scores (p < .001). Compared to non-ambulant individuals (Mean = 24.35), transitioning and ambulant individuals scored significantly higher: +11.23 (SE = 0.64, t(463) = 17.50, p < .001) and +13.84 (SE = 0.85, t(463) = 16.32, p < .001), respectively.

The same pattern was observed across functional domains. For the shoulder domain (reference Mean = 3.47), transitioning individuals scored +5.55 (SE = 0.29, p < .001) and ambulant individuals +7.01 (SE = 0.35, p < .001). For the elbow domain (reference Mean = 9.90), scores were +4.93 (SE = 0.34, p < .001) and +5.88 (SE = 0.45, p < .001), though the difference between transitioning and ambulant was marginal (p = 0.05). For the distal domain (reference Mean = 10.74), differences were +1.06 (SE = 0.12, p < .001) and +1.45 (SE = 0.17, p < .001), respectively.

Post-hoc tests confirmed significant pairwise differences across all groups for each domain (p < .0001), except between transitioning and ambulant in the elbow domain.

Table 2 shows baseline PUL 2.0 scores subdivided by entry item.

**Table 2.** Baseline PUL 2.0 scores subdivided by entry item. Key to table: *N* *=* *number of patients; number of assessments,* P-value was calculated via linear mixed model accounting for repeated measures, this was calculated only among subgroups with >10 individuals per groups; **=overall significant difference among the subgroups; post-hoc comparisons were conducted using estimated marginal means. Groups that share the same letter(s) are not significantly different from each other at the chosen significance level (e.g., p < 0.05).

		PUL 2.0 Entry item						
		1	2	3	4	5	6	p-value
Non ambulant	Total	13.1 (11.0, 15.3)^a^ N = 37;94	16.1 (13.6, 18.7)^b^ N = 24;36	19.6 (17.5, 21.6)^c^ N = 46;73	27.4 (24.7, 30.1)^d^ N = 23;29	31.4 (29.2, 33.6)^e^ N = 42;63	35.4 (32.9, 38.0)^f^ N = 28;38	<0.001**
	Shoulder	0.2 (0.2, 0.2)^a^ N = 37;94	0.3(0.3, 0.3)^a^ N = 24;36	0.5(−0.2, 1.2)^a^ N = 46;73	4.2 (3.2, 5.2)^b^ N = 23;29	6.5 (5.8, 7.3)^c^ N = 42;63	9.3 (8.4, 10.2)^d^ N = 28;38	<0.001**
	Elbow	3.9 (2.7, 5.1)^a^ N = 37;94	5.7 (4.2, 7.1)^a^ N = 24;36	8.4 (7.2, 9.6)^a^ N = 46;73	12.0 (10.5, 13.6)^b^ N = 23;29	13.6(12.4, 14.8)^c^ N = 42;63	14.5 (13.0, 16.0)^d^ N = 28;38	<0.001**
	Distal	9.1 (8.3, 9.9)^a^ N = 37;94	10.1 (9.2, 11.0)^b^ N = 24;36	10.6 (9.9, 11.4)^b,c^ N = 46;73	11.1 (10.2, 12.1)^b,c,d^ N = 23;29	11.3 (10.5, 12.1)^c,d^ N = 42;63	11.7 (10.8, 12.6)^d^ N = 28;38	<0.001**
Transitioning	Total	– N = 0	– N = 0	23 (23.0, 23.0) N = 1;1	30.4 (22.1, 38.8) N = 4;5	33.8 (30.4, 37.2)^a^ N = 24;32	38.5 (36.4, 40.7)^b^ N = 72;113	<0.001**
	Shoulder	– N = 0	– N = 0	0 (0.0, 0.0) N = 1;1	5.0 (0.6, 9.3) N = 4;5	7.2 (5.5, 9.0)^a^ N = 24;32	10.1 (9.0, 11.1)^b^ N = 72;113	<0.001**
	Elbow	– N = 0	– N = 0	11.0 (11.0, 11.0) N = 1;1	14.4 (10.3, 18.5) N = 4;5	14.9 (13.2, 16.6)^a^ N = 24;32	16.1 (15.1, 17.2)^a^ N = 72;113	<0.001**
	Distal	– N = 0	– N = 0	12 (12.0, 12.0) N = 1;1	11.1 (9.6, 12.5) N = 4;5	11.6 (11.1, 12.2)^a^ N = 24;32	12.3 (12.3, 12.7)^b^ N = 72;113	<0.001**
Ambulant	Total	– N = 0	– N = 0	– N = 0	– N = 0	36.1 (32.5, 39.7)^a^ N = 11;14	40.1 (38.9, 41.4)^b^ N = 80;186	<0.001**
	Shoulder	– N = 0	– N = 0	– N = 0	– N = 0	8.3 (6.2, 10.3)^a^ N = 11;14	11.1 (10.4, 11.8)^b^ N = 80;186	<0.001**
	Elbow	– N = 0	– N = 0	– N = 0	– N = 0	15.8 (14.3, 17.3)^a^ N = 11;14	16.6 (16.1, 17.1)^a^ N = 80;186	<0.001**
	Distal	– N = 0	– N = 0	– N = 0	– N = 0	12.0 (11.4, 12.6)^a^ N = 11;14	12.4 (12.2, 12.6)^a^ N = 80;186	<0.001**

### PUL 2.0 changes

In the overall cohort of 219 individuals/684 assessments, the mean 36-month changes were −7.81 (95% CI = −8.26, −7.36) for the total scores, −3.22 (95% CI = −3.45, −2.98) for the shoulder domain, −3.52 (95% CI = −3.77, −3.28) for the elbow domain, and −1.06 (95% CI = −1.17, −0.96) for the distal domain. A linear mixed model revealed a statistically significant main effect of time for all outcomes (p < .0001). Post-hoc pairwise comparisons showed significant differences between all timepoints (baseline vs 12, 24, and 36 months; 12 vs 24 and 36 months; 24 vs 36 months; all p < .0001).

### PUL 2.0 changes and functional status

The largest 36-month changes in the total scores were observed in the transitioning cohort (−11.62 points, 95% CI = −12.40, −10.84) with smaller changes observed in the non-ambulant (−8.09, 95% CI = −8.66, −7.51) and ambulant (−4.51, 95% CI = −5.23, −3.80) subgroups.

At shoulder level, the largest 36-month changes were observed in the transitioning cohort (−5.85, 95% CI = −6.26, −5.433) with smaller changes observed in the non-ambulant (−2.38, 95% CI = −2.68, −2.07) and ambulant (−2.59, 95% CI = −2.97, −2.20) subgroups.

At elbow level, the largest 36-month changes were observed in the transitioning cohort (−4.80, 95% CI = −5.22, −4.37) with smaller changes observed in the non-ambulant (−4.20, 95% CI = −4.52, −3.90) and ambulant (−1.50, 95% CI = −1.90, −1.11) subgroups.

At distal level, the largest 36-month changes were observed in the non-ambulant group (−1.5, 95% CI = −1.63, −1.35) with smaller changes observed in the transitioning (−0.98, 95% CI = −1.17, −0.80) and ambulant (−0.42, 95% CI = −0.60, −0.25) subgroups.

**Figure 1.**
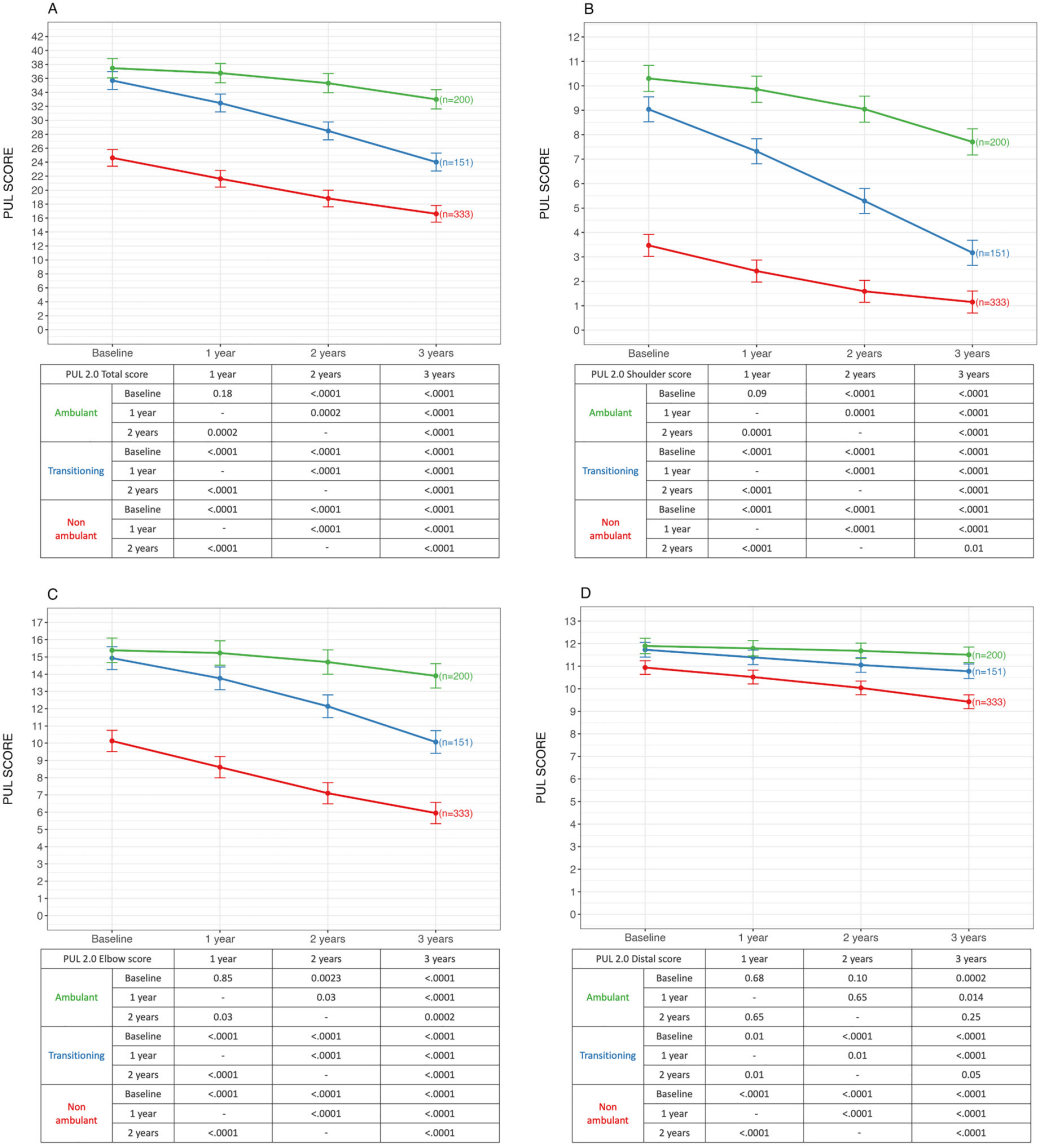
PUL 2.0 changes according to ambulatory status by domain. Key to figure: Line represent mean, error bars represents 95% confidence intervals. Panel A = Total score, Panel B = Shoulder domain, Panel C = Elbow domain, Panel D = Distal domain; Green = Ambulatory patients, Blue = transitioning patients, Red = Non ambulatory patients. P-value was calculated via linear mixed model accounting for repeated measures, this was calculated only among subgroups with >10 individuals per groups; post-hoc comparisons were conducted using estimated marginal means (P value adjustment: tukey method). P-values are shown in the tables below each panel.

Figure 1 shows overtime changes in the whole cohort subdivided by ambulatory status.

### PUL 2.0 changes and entry criteria

The largest 36-month changes in the total scores were observed in the subgroup with entry score of 4 (−11.97, 95% CI = −13.48, −10.46), followed by entry scores of 5 (−11.55, 95% CI = −12.46, −10.63) and 6 (−8.72, 95% CI = −9.32, −8.12) with smaller changes observed in those with entry scores of 3 (−4.79, 95% CI = −5.88, −3.70), 2 (−2.19, 95% CI = −3.70, −0.68) and 1 (−2.95, 95% CI = −4.06, −1.84).

**Figure 2.**
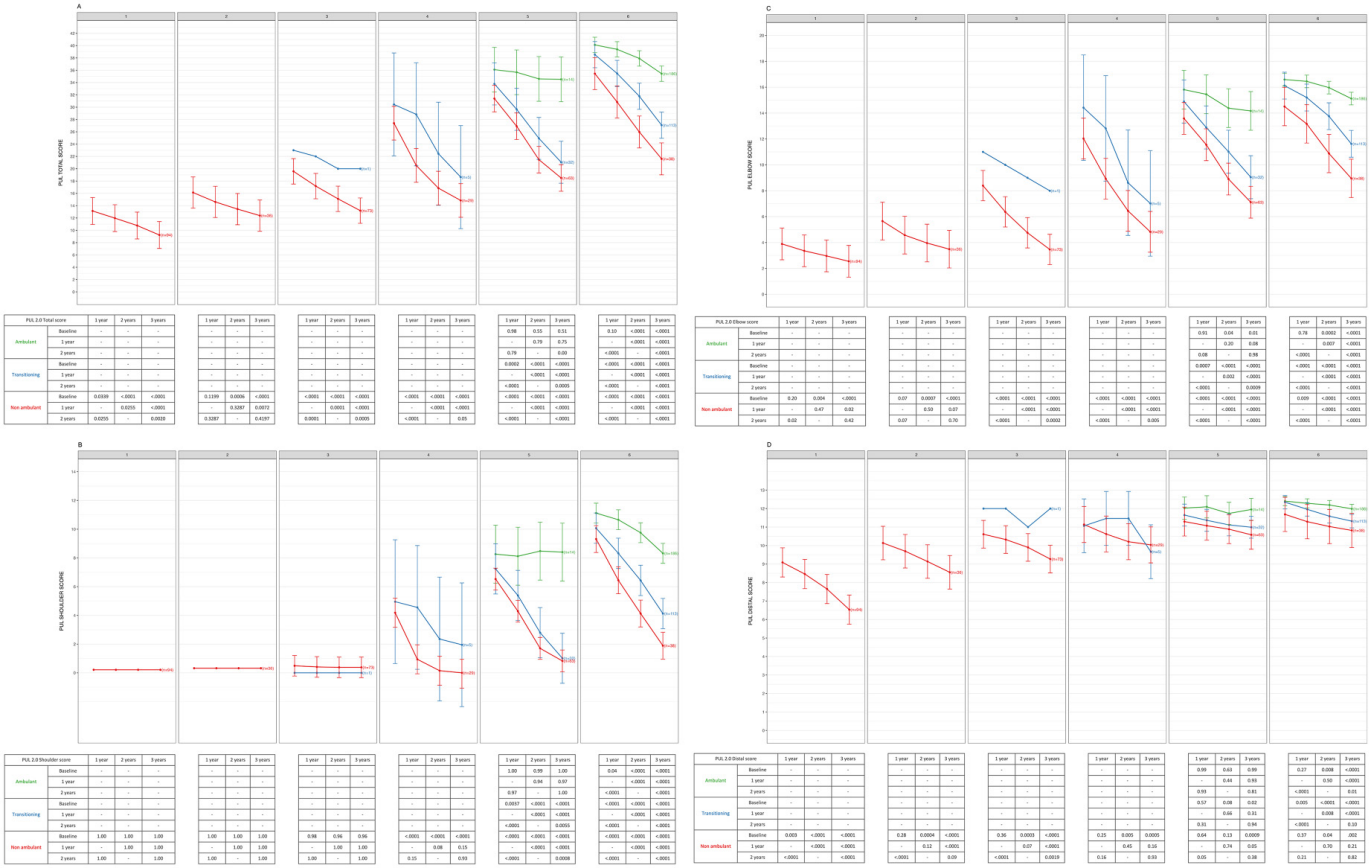
36 months PUL 2.0 changes subdivided by entry item and ambulatory status. Key to figure: Line represent mean, error bars represents 95% confidence intervals. Panel A = Total score, Panel B = Shoulder domain, Panel C = Elbow domain, Panel D = Distal domain; Green = Ambulatory patients, Blue = transitioning patients, Red = Non ambulatory patients. P-value was calculated via linear mixed model accounting for repeated measures, this was calculated only among subgroups with >10 individuals per groups; post-hoc comparisons were conducted using estimated marginal means (P value adjustment: tukey method). P-values are shown in the tables below each panel.

Figure 2 shows details of the changes according to functional status and entry criteria.

## Discussion

The findings from the 36-months longitudinal analysis confirm and expand the clear trend of functional decline over time across 12, 24, and 36 months. Overall, the total score showed significant decline, with an average decrease of 2.38 points at 12 months, 5.02 points at 24 months, and 7.78 points at 36 months. The differences between baseline and each time point (12, 24, and 36 months) were highly significant (p < .0001), and the differences between the time intervals (12–24 months, 12–36 months, and 24–36 months) were also significant (p < .0001). This progressive deterioration underscores the challenges faced by patients over time, reflecting a substantial loss of functional abilities. Our data also confirm the large variability of changes previously reported on the PUL 2.0 and on other measures,^4,20,35–44^ highlighting the need for the identification of variables that may identify more accurate trajectories of progression within the whole cohort. Recent clinical trials using the PUL 2.0 as primary or secondary outcome have used the PUL 2.0 entry item as an inclusion criteria.^45–48^ Based on the previously published natural history studies,^17,18^ only patients with entry item between 3 and 5 were included as they have more chances of showing a decline in the 12 month time frame of the trial.

Our new findings with 36 month follow up expand previous findings and confirm that the entry item score at baseline plays a pivotal role to identify different trajectories of progression. Over 36 months a decline was observed in all the subgroups irrespective of the entry item, but the magnitude of changes was different. Patients with the lowest entry items (1 and 2) have already lost most of or all the shoulder and elbow scores and the distal domain shows a slower decline, as also suggested by MRI studies reporting minimal changes in the distal muscles.^23,49,50^ The largest changes were observed in boys with an entry item of 5 and 4 who have relatively preserved shoulder function. Both showed a decline in both shoulder and elbow domains with those with an entry item of 5, who had higher scores on shoulder at baseline, having the largest changes over three years. Boys with an entry item of 6 who have less involvement of the proximal muscles, as also observed on MRI,^23,49,50^ tend to be more stable on a short term follow up but they also show decline over 36-months.

While these findings already provide useful information on possible trajectories according to entry item, each of the entry item subgroups still shows some variability in changes and better information can be achieved if we also consider functional status. As already suggested in our study assessing PUL 2.0 changes at 2 years in relation to functional status, in the present study we confirm the significant effect of ambulatory status on total,shoulder, elbow, and distal scores at 36-months. Transitioning boys, i.e., those who lost ambulation during the 36-months follow up, were the ones who showed the largest changes on total scores, this reflecting the largest changes also observed in shoulder and elbow domain. It is of note that even if in the ambulant group the mild but progressive decline was mainly driven by the shoulder scores, in the third year we also noticed some initial decline in elbow scores, that was not observed in the first two years. A decline in distal domain in contrast was mainly observed in the non-ambulant boys.

Our study was initially also aimed to analyze if the type and regime of steroid used (corticosteroids vs. prednisone) could further contribute to identifying the trajectories in our cohort. However, the possibility to perform a meaningful analysis was limited by the large discrepancy between the number of patients treated with prednisone (5%) and deflazacort (95% of the treated ones). The analysis was further limited by evidence that patients in the naïve cohort were nearly exclusively non-ambulant and in the non-ambulant cohort the dose/kg of steroids was very variable. As detailed information on duration of treatment and exact dose were not easily available, the steroid data were not included in the final analysis.

Although collected at a national level, when stratified using two or more criteria, the number in some of the subgroups was relatively small, this restricting the possibility of a more complex analyses.^17^ Further studies combining data from other networks could allow a larger dataset and the use of machine learning or other new approaches assessing the combination of multiple variables to identify more defined trajectories of progression and their predictors.

Even with these limitations, our findings provide long term data for the PUL 2.0, underscoring the multifaceted influences of ambulatory status, and baseline values. Our new findings, expanding the data available at 12 and 24 months and providing reference data also for 36-months changes, also provide additional information on the combination of functional status and entry criteria, that is consistently being used in clinical trials as one of the inclusion/exclusion criteria for the stratification of the cohorts. The transitioning group, that includes boys who are still ambulant at baseline but have relatively low NSAA scores (mean 16) or 6MWT (mean 313 meters), who generally have entry items of 4 and 5 appears to be particularly prone to changes not only in the gross motor (loss of ambulation), but also showing the largest changes in the upper limb function both on short term and long term follow up. Further prospective studies will help to better delineate functional cut off criteria for the transition group that in this retrospective study could only be defined by their outcome, i.e., having lost the ambulation within the duration of the 36-months observation. These data will be of help at the time of designing new trials in cohorts including transitioning or non-ambulant patients and, following the recent approval of new therapies for non-ambulant patients, will provide a reference for comparison with the long term results in treated patients. The availability of both total scores and scores for individual domains will also provide the opportunity to have more specific comparison both for selected groups in whom one domain may be more relevant or for larger cohorts in whom the total scores will reduce the possibility of floor and ceiling effect.
